# Progesterone and Bone: Actions Promoting Bone Health in Women

**DOI:** 10.4061/2010/845180

**Published:** 2010-10-31

**Authors:** Vanadin Seifert-Klauss, Jerilynn C. Prior

**Affiliations:** ^1^Frauenklinik der Technischen Universität München (TUM), Klinikum Rechts der Isar, Ismaninger Str., 22 81675 Muenchen, Germany; ^2^Division of Endocrinology and Metabolism, Department of Internal Medicine, University of British Columbia and Centre for Menstrual Cycle and Ovulation Research (CeMCOR), 2775 Laurel Street, Vancouver, BC, Canada V5Z 1M9

## Abstract

Estradiol (E_2_) and progesterone (P_4_) collaborate within bone remodelling on resorption (E_2_) and formation (P_4_). We integrate evidence that P_4_ may prevent and, with antiresorptives, treat women's osteoporosis. P_4_ stimulates osteoblast differentiation *in vitro*. Menarche (E_2_) and onset of ovulation (P_4_) both contribute to peak BMD. Meta-analysis of 5 studies confirms that regularly cycling premenopausal women lose bone mineral density (BMD) related to subclinical ovulatory disturbances (SODs). Cyclic progestin prevents bone loss in healthy premenopausal women with amenorrhea or SOD. BMD loss is more rapid in perimenopause than postmenopause—decreased bone formation due to P_4_ deficiency contributes. In 4 placebo-controlled RCTs, BMD loss is not prevented by P_4_ in postmenopausal women with increased bone turnover. However, 5 studies of E_2_-MPA co-therapy show greater BMD increases versus E_2_ alone. P_4_ fracture data are lacking. P_4_ prevents bone loss in pre- and possibly perimenopausal women; progesterone co-therapy with antiresorptives may increase bone formation and BMD.

## 1. Introduction

Osteoporosis has been considered primarily because of estrogen deficiency at menopause since Fuller Albright [1]. Most scientists view estradiol as women's sole bone-active gonadal steroid. In reality, estradiol and progesterone work together in every tissue in women's normal physiology [2]. Estrogen plays positive roles in bone biology and osteoporosis prevention and treatment primarily through decreasing bone resorption [3–5]. There is also compelling evidence that powerful bone-destructive cytokines such as IL-1, IL-6, and TNF*α* are released and increase rapidly with dropping estradiol levels, as occurs with surgical menopause [6]. Estradiol achieves its positive bone effects largely through two key actions: facilitation of vitamin D-related intestinal calcium absorption [4, 7] and suppression of bone resorption through the osteoprotegerin/RANK/RANKL system [7]. It is also clinically obvious that premenopausal women with amenorrhea have lower estradiol levels and lower bone mineral density (BMD) and/or lose bone rapidly [8]. 

Not until recently did randomized, placebo-controlled trial data from the WHI studies show that treatment with conjugated equine estrogen (CEE) plus medroxyprogesterone (MPA) or with CEE alone (in women with hysterectomy) prevented osteoporotic fractures in asymptomatic postmenopausal women ages 50–79 [5, 9]. Estradiol's role in human bone health is unmistakable. However, progesterone is usually a present, but an unrecognized partner in bone. With amenorrhea and surgical or natural menopause, not only are estradiol levels low or dropping, progesterone levels are also low. While, in these conditions, estrogen and progesterone deficiency are nearly indistinguishable, progesterone deficiency precedes low estradiol levels in perimenopause [10], for example, and with ovulatory disturbances, occurs silently in regular cycles with normal estrogen levels [11].

The purpose of this paper and meta-analyses is to study recent clinical evidence that endogenous progesterone plays a role in bone health. So far, three *in vitro* publications document progesterone's ability to increase osteoblast numbers [12–14] as well as its effects to promote osteoblast maturation and differentiation [13]. Progesterone appears to play a differing but also physiological role in partnership with estrogen in achieving optimal peak bone mass. Medroxyprogesterone increases premenopausal spine BMD as physiological-dose cyclic therapy in a randomized controlled trial (RCT) for healthy women experiencing hypothalamic amenorrhea, oligomenorrhea, anovulation, or short luteal phase cycles [15]. 

Progesterone may also have a *therapeutic* role in postmenopausal osteoporosis if paired with an antiresorptive therapy. Thus this paper highlights the accumulating human evidence for a role of progesterone for increasing bone formation in estrogen-replete women with regular menstrual cycles. 

From a teleological point of view, a higher trabecular bone mass in women is needed in preparation for building of the fetal skeleton during pregnancy. Interestingly, the third trimester of pregnancy, during which 80% of the fetal skeleton is mineralized, coincides with the maximum rate of progesterone production in human physiology. Under normal circumstances, enough trabecular bone has been accumulated and maintained in women's skeletons to serve as a reservoir for the calcium needs of both mother and fetus during the months of pregnancy, and for the infant during potential months of breast-feeding. The fact that bone morphogenic proteins play a crucial role in both ovulation and bone metabolism points towards a functional link between bone and reproductive systems aimed at preparing for the increased demands of pregnancy. 

Knowledge of progesterone's actions in the context of the latest genetic, receptor, and bone ligand systems is in its infancy—relationships may well exist between progesterone and the immune system through osteoblast and hematopoietic stem cell interactions in bone marrow [16], through progesterone's known brain anti-inflammatory and antiapoptotic actions [17], and through potential relationships with emerging bone-related molecules such as sclerostin, vascular endothelial growth factor (VEGF), and basic fibroblast growth factor (bFGF), to name a few. These molecular biology issues, however, are beyond the scope of this primarily clinical and therapeutic review. 

Over the last 20 years [14], a number of controlled trials and prospective studies suggest that progesterone may have a role in treatment of pre- or perimenopausal women with regular, estrogen-sufficient menstrual cycles who, however, are also experiencing ovulatory disturbances (anovulation, or short luteal phase length cycles). The most prevalent of abnormal cycles are *subclinical *ovulatory disturbances (SOD) that are unremarkable because they occur within regular, asymptomatic menstrual cycles [11, 18]. They have an increased incidence in normal weight women with subclinical cognitive dietary restraint [19], women working shifts and in stressful environments. However, currently there are no published data about effects of progesterone on human bone architecture and bone quantitative histomorphometry in either the cortical or cancellous bone compartments, or about the potential of molecularly identical progesterone to decrease fracture risk. 

## 2. Materials and Methods

Studies on endogenous and/or physiologic progesterone concentrations and bone are very scarce: a PubMed search carried out in January, 2010 using the MeSH terms “endogenous progesterone and bone” yielded 51 results since 1975. Similarly, 83 papers since 1968 were found using the terms “physiological progesterone and bone.” We excluded all citations concerning animals and/or nonhuman cell lines (most of which have been previously reviewed [14]), those relating to preterm infants, and publications on synthetic androgenic or estrogenic progestins, depot-MPA or other injectable progestins, or those in supraphysiological doses. This paper will focus on the physiological and pharmaceutical actions of progesterone and/or “physiological dose” medroxyprogesterone acetate (MPA) as the progesterone-derived therapy most commonly prescribed in the USA and Canada. It is important to note that, when using the word, “progesterone,” we are always discussing the native human steroid. 

If reference to the actions of a progestin or progestogen is required, this paper will specifically state the compound involved. NETA—and other progestins that primarily are metabolized to estrogen or that act through androgen osteoblast receptors—is not covered because our focus is on physiological bone actions of the human steroid, progesterone. 

Given our broad purpose, we are evaluating data from diverse sources; we are also, of necessity, comparing studies with differing methodologies and designs. Therefore, although numerical summaries are created where possible, we have not subjected these combined data to statistical analysis. 

## 3. Results and Discussion

### 3.1. Progesterone and Bone Formation in Osteoblasts

**Figure 1:**
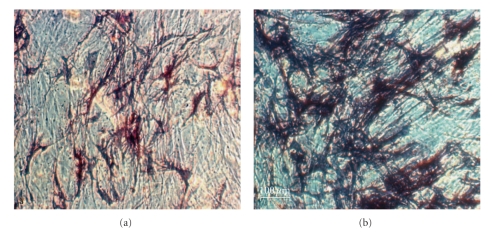
This photomicrograph (at 400 power magnification) shows human osteoblasts in culture after 28 days stained to show Alkaline Phosphatase production as dark blue. (a) Estradiol at a physiological concentration. (b) Estradiol alone for 7 days combined with Progesterone for 21 days. Note the lack of alkaline phosphatase staining in (a) exposed to estrogen alone, and the marked ALP staining indicating osteoblast differentiation/maturation induced by the addition of progesterone, (b) This figure is reprinted from [20] with permission from authors (Schmidmayr M and Seifert-Klauss V). Publisher permission provided.

Most studies of the action of progesterone on human osteoblasts *in vitro* have assessed effects over a maximum of only 72 hours' duration [12–14]. One recent study from Munich, however, used long-term cultures of human osteoblasts (HOBs) to characterize the influence of progesterone and estradiol on bone proliferation (using a hexosaminidase assay) and differentiation (using alkaline phosphatase [ALP] staining). This study quantified ALP production photometrically with extinction at 405 nm following incubation with p-Nitrophenyl-Phosphate (pNPP) and buffer [20] (Figure 1). These primary osteoblast cultures, derived from nonosteoporotic perimenopausal women undergoing hip replacement surgery, were exposed to 7 or 21 days of progesterone with and without estradiol pre- and cotreatment [20]. 

The effect of progesterone *in vitro o*n differentiation of osteoblasts was dose-dependent for progesterone [20], independent of estradiol, and reached its maximal stimulation at concentrations of 10^−9^ M progesterone (Figure 1). This progesterone level corresponds with luteal phase serum progesterone levels in ovulatory cycles. Seven days of exposure to physiologic levels of progesterone (6.4 × 10^−7^–10^−9^ M) led to increased ALP concentrations of 70% (*P* = .004–.019), while a *supraphysiological* progesterone concentration (6.4 × 10^−6^ M) caused a significant 50% reduction in ALP (*P* = .028). After 21 days of physiological progesterone exposure, the ALP production increased 2.7-fold (*P* = .000 to  .004). At supraphysiological progesterone concentrations ALP staining decreased by 80% (*P* = .03). Thus there was a physiological osteoblast differentiation dose-response curve optimal at luteal phase levels with suppression at high progesterone doses. In contrast to expectation and the observations of others [13], this effect was independent of pre- or cotreatment with estradiol. Proliferation, however, was not significantly affected by progesterone in the absence of estradiol [20]. These results suggest an osteoanabolic function of progesterone, while showing for the first time that supraphysiological progesterone concentrations suppressed osteoblast differentiation. These data are the longest of any *in vitro *data on human osteoblasts and progesterone. They clearly show a progesterone osteoblast differentiation dose response and independence from estradiol.

### 3.2. Progesterone and Bone within Menstrual Cycles, Relation to Peak Bone Mass, and Premenopausal Bone Loss Prevention

#### 3.2.1. Progesterone and Bone Remodeling in the Menstrual Cycle

During the bone remodeling cycle within a single bone multicellular unit (BMU), activation is followed by increased resorption, which in turn is followed by osteoid formation and osteoid mineralization [21]. Within a single BMU, formation takes approximately two to three weeks while formation and initial (incomplete) mineralization requires at least three months [21]. Perhaps in compensation, osteoblasts appear to be more abundant and also more plastic than osteoclasts and evolve to become both lining cells and osteocytes [22]. 

Although a number of studies have been performed of bone turnover markers across the menstrual cycle [14], most of them used less precise or specific markers and methods, inadequately differentiated ovulatory from anovulatory cycles (by hormonal measures), or recorded too few cycles to be helpful. Some studies with careful cycle bone marker documentation are now available [23, 24] and tend to show increased follicular phase urinary markers of bone resorption in addition to increased luteal phase markers of bone formation. Chiu et al. found the bone resorption marker deoxypyridinoline (D-Pyr) to be higher during the follicular phase than in the luteal phase and to correlate negatively both with E_2_ values measured 6 and 8 days earlier and with progesterone levels measured 2–6 days earlier [23]. Unfortunately, the authors did not differentiate between ovulatory and anovulatory cycles, which judging from the wide span of progesterone values must have been mixed in their study. They concluded that “normal women experience monthly episodes of increased bone resorption from menarche to menopause” [23]. In another study, 10 Japanese women with normal ovulatory function showed significant decreases in CTX, free D-Pyr, and serum intact carboxyterminal telopeptide (ICTP) during the luteal phase and significantly higher serum PTH levels during the follicular than the luteal phase [25], perhaps because of coupling of resorption and formation. Caufriez et al. very recently proposed a potential role of endogenous progesterone in modulation of GH-secretion (along with prolactin and TRH) during the normal menstrual cycle [26]. In their study of 10 young Belgian women, 24-hour growth hormone secretion was associated with higher progesterone levels, and daytime GH secretion was increased in the luteal phase compared with the follicular phase [26]. Another European group had already found that PTH concentrations were highest on day three of the menstrual cycle, but had not monitored ovulation and found no relation to progesterone levels [27]. Earlier, a Danish serial serum hormone study in eight healthy women aged 20–47 found osteoblastic activity to be higher during the well-documented luteal phase by measurements of osteocalcin (OC) and bone-specific alkaline phosphatase (BAP) [24]. In addition, this study also observed the highest level of IGF-1 (then called somatomedin C) during the luteal phase of the menstrual cycle [24], with which the recent growth hormone data agree [26]. Thus several studies confirm higher follicular phase bone resorption rates and higher luteal phase rates of bone formation.

#### 3.2.2. Progesterone, Ovulation, and Peak Bone Mass

Young women gain body size (BMI), bone size, and BMD rapidly around the time of peak height velocity and menarche [29, 30]. Although not well characterized, it is known that levels of estradiol, testosterone, and growth hormone are increased during this period of bone growth and reproductive maturation. However, ovulatory cycles are rare at menarche and become more prevalent only with increasing time since the first period [31, 32]. There are sparse data about relationships between bone change and ovulation, documented either by Tanner breast stages (in which Tanner stages 4 and 5 indicate the presence of progesterone [33]) and/or by hormonal measures. 

**Figure 2:**
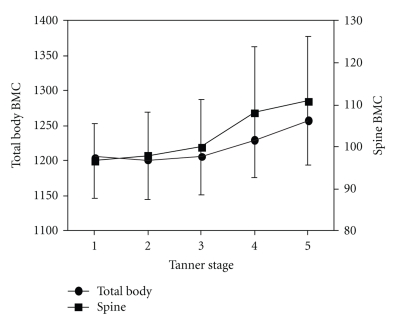
This diagram illustrates changes in Total Body (black circle) and Spine (black square) Bone Mineral Content (BMC) adjusted for body size in a population-based cohort of adolescents (mean 11.8 years old) by Tanner Stages on the *X*-axis. It is drawn from data in Table 3 [28]. Endocrine Society permission provided.

A recent population-based study of estradiol receptor polymorphisms and BMC data (corrected for bone area but not BMI or body size) compared BMC with breast Tanner Stage in a cross-sectional study of girls who averaged aged 11.8. These data showed that bone size-adjusted BMC is greater in Tanner Stage 5 than in Stage 1 (Figure 2) [28]. 

Such cross-sectional population-based data fit with prospective observations in the Teen Bone Study [30], a prospective, observational study in a convenience cohort of 38 girls aged 9–11 (mean initial age 10.6 ± [SD] 0.6 years) that documented total body and spine BMD and BMC at baseline and yearly. In addition six monthly measurements were made of weight, height, seated height, wrist width, BMI, and questionnaires about calcium intakes and exercise [30]. These young women were also examined every six months for pubertal maturation. The onset of menstruation, menstrual cycle calendar data, and weekly salivary progesterone levels were used to assess the prevalence of ovulation based on a threshold value of higher than 40 nmol/L [34, 35]. 

**Figure 3:**
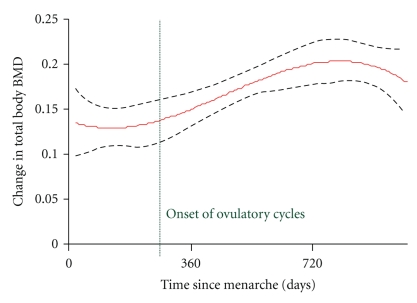
This graph shows the multivariable regression for the mean and the 95% confidence interval of the change in total body bone mineral density (BMD) over 3 years in relationship to time since menarche in 38 peripubertal girls studied prospectively [30]. The vertical line shows the earliest, in a subset of 13 girls who provided menstrual calendar data and salivary progesterone levels, that ovulation could be diagnosed [34]. Reprinted with permission of the authors. Society for Bone and Mineral Research permission provided.

Menarche occurred for 33 young women during the course of the 3-year study [34]. Based on 93 menstrual cycles from 13 young women that averaged 36 days long (range 20–119) and their weekly salivary progesterone data (*n* = 163 samples), 27 (29%) cycles were ovulatory while 66 (71%) were anovulatory. Ovulation was documented no earlier than 10 ± 5 months postmenarche [34].  Figure 3 shows that total body BMD increased significantly by the number of days since menarche (day 0 in Figure 3); in particular BMD increased more following the onset of ovulation [34]. Gains in bone density were greater 10 ± 5 months after menarche following which time ovulation first developed (shown with the dotted vertical line) than before (*r* ^2^ = 0.40, *P* < .0001). It is known that pubertal bone gain strongly relates to body size and weight. Despite the observation that BMI increased with time following menarche, no significant relationship between changes in BMI and changes in spine BMD or BMC was found. However, changes in BMI did significantly relate to changes in total body BMC (*r* = .421, *P* = .001). In summary, these prospective teen bone and ovulation data, although limited, suggest that progesterone adds to the bone gains of menarche. Following the onset of ovulation (that is delayed on average for almost a year after menarche) bone gain is greater than early after menarche suggesting that progesterone contributes to a high ideal peak bone mass in adolescent girls [36].

#### 3.2.3. Menstrual Cycle Disturbances and Bone (Amenorrhea and Oligomenorrhea)

Absence of menstruation following menarche is associated with low levels of both estradiol and progesterone, whether related to hypothalamic suppression (usually due to calorie insufficiency for the level of energy expenditure, emotional/social stressors, or physical illness) or to ovarian dysfunction (such as Turner's Syndrome or other causes of premature menopause). In general, longer cycle lengths are associated with lower estradiol levels and BMD values [37, 38]. In some young women, however, oligomenorrhea is related to anovulatory androgen excess (AAE, as in “polycystic ovary syndrome,” PCOS) and associated with higher LH, testosterone, DHEAS, androstenedione, and estrogen levels, but absent or rare ovulation and absolute or relative progesterone deficiency [39]. Because these oligomenorrheic, hyperandrogenic young women likely have different changes in bone compared to regularly cyclic or to amenorrheic women, and their prospective bone changes are not well characterized, we will not review AAE/PCOS further here. 

Epidemiological data suggest that primary amenorrhea is rare in the population (about 0.1%) [40]; secondary amenorrhea is also uncommon (<1.3%) in the premenopausal population, although it is more prevalent in teen-aged population-based samples [41, 42]. Both hypothalamic amenorrhea and oligomenorrhea are associated with significantly lower BMD values as well as lower FSH levels [43]. Furthermore, there is rapid bone loss after the onset of amenorrhea [44]. However, with longer durations of amenorrhea (more than three years), bone turnover and bone loss both appear to decrease [36] while absolute BMD values remain low. Thus, among premenopausal women, long cycles associated with hypothalamic oligomenorrhea and amenorrhea are both risk factors for bone loss and low BMD.

#### 3.2.4. Ovulatory Disturbances (Anovulation/Short Luteal Phases) and Bone in Regular Cycles

Regular cycles with normal estradiol levels may vary in their progesterone characteristics. Such cycles may be normally ovulatory, anovulatory or have short luteal phase lengths that result in decreased total progesterone production [11]. Subclinical ovulatory disturbances (SODs, meaning regular cycles with either anovulation and short luteal phase lengths) may pose a risk for bone remodelling imbalance and bone loss despite regular, estrogen-sufficient menstrual cycles. 

Currently five published observational cohort studies in a total of 458 women have prospectively examined menstrual cycles by ovulatory characteristics and change in BMD [11, 45–48] measured by dual energy X-ray absorptiometry measures (DXA) or spinal quantitative computed tomo-graphy (QCT). These studies span one to four years in healthy, largely Caucasian premenopausal women (ages 20–42) not using oral contraceptives (OCs) or other bone-active therapies. Documentation of luteal phase lengths used Quantitative Basal Temperature (QBT) methods (validated against the serum LH peak [49] and daily urinary progesterone excretion by pregnanediol (PdG), resp. [50]). 

Ovulatory characteristics are variously described in the five studies.  Table 1 shows the similarities and differences among these published studies—all are in primarily well-educated, white women who average >10 years since menarche and are mainly in their 30s (mean age 31.4) except for the younger women (mean age 22.1) in the Bedford study [48]. 

In these five studies assessing prospective bone change by the incidence of ovulatory disturbances, the total number of cycles with documentation of ovulation (Table 1) varied from a median of 10/year in the Prior 1990 study to 5/year in the Waugh investigation, to 6.8/year in the Bedford study, to 2.7/year in the Waller, and 1.5/year in the Prior 1996 data. In this latter four-year follow-up study, women collected menstrual cycle and QBT data for 3–6 months at the end of the fourth year and before repeat BMD measurement at the five-year anniversary of their initial QCT. Here, the median number of cycles of data collected by the 27 reported women was 6 (range 3–46) with a minimum of 3 cycles [45]. 

In those three studies that had documentation of ovulation in at least five cycles/year, which reached a total of almost 400 women, those with more prevalent normally ovulatory cycles had a +1.23% gain versus the −1.00% loss/year in those with ovulatory disturbances (Table 1) [11, 46, 48]. By contrast, studies with fewer cycles between bone density measurements [45] or few measurements not within the bone change window [47] were not able to show any such ovulation-bone change relationship [45, 47]. However, the luteal index (mean luteal phase length divided by mean cycle length) from year one of the Prior study [11] continued to relate positively (*r* = 0.339. *P* = .043) to the entire five-year bone change [45]. Also in the total body bone density reported by Waller, normally cycling women experienced a +0.02% change while those with ovulatory disturbances experienced a –1.7% loss (*P* = .08) [47]. Furthermore, the Bedford study showed that total hip BMD change, in addition to spinal BMD, was significantly related to ovulatory disturbances (−0.6% versus +0.9%, *P* = .001) [48]. In summary, it appears that five or more cycles of ovulation-documented data per year are needed to “see” any bone change related to progesterone production within regular menstrual cycles. 

Several cross-sectional studies have also addressed the issue of ovulation and BMD. The most influential of these has a nested case-control design within a population-based sample (The Michigan Bone Health Study) [54]. A randomly sampled cohort of premenopausal women ages 25–45 (*n* = 582) all had BMD measured by DXA. Those in the lowest 10th percentile of bone density (cases) and those in the 50th to 75th percentile of BMD (controls), who had regular cycles, were on no hormones (*n* = 31 cases and 34 controls) collected daily first morning urines for LH, FSH, and excretory products of estrogen (E1C) and progesterone (PdG) over two cycles or 84 days [54]. Cases (women with low BMD) were smaller and leaner than controls (BMI 23.6 versus 26.1), probably of lower socioeconomic status (based on significantly fewer years of education) and were less likely to use alcohol or to have taken oral contraceptives [28]. 

Results of this cross-sectional study showed lower PdG and E1C excretions across cycles in cases compared with controls. All three summary measures of PdG were lower in cases (peak, mean, and area under the curve with *P*  = .002–.006). E1C was also lower (with *P* = .01 to  .008). Although not statistically significant, anovulation rates were higher in cases (14.8%) than in controls (8.8%). The data suggest that lower BMD values are related to subtle disturbances in ovulation and perhaps estradiol levels within regular cycles. 

The final two cross-sectional studies failed to confirm a relationship between ovulatory disturbances and bone change in premenopausal women [51, 53]. These studies typically have measured ovulatory function in only one cycle and/or did not document short luteal phase lengths that are the most prevalent subclinical disturbances of ovulation [51, 53]. It is likely that a cross-sectional study does not have the power to show a bone-ovulation relationship because of the great within-woman variability of subclinical ovulatory disturbances.

#### 3.2.5. Progesterone Therapy for Premenopausal Bone Loss Prevention

If the above associations of ovulatory disturbances with less positive changes in bone hold true, women with subclinical ovulatory disturbances who are currently undiagnosed and overlooked as having bone risks might be experiencing bone loss over many asymptomatic premenopausal years. 

Data on progesterone's osteoblast-differentiation effects suggest that luteal phase “progesterone replacement” may be an effective treatment for SOD. So far, no progesterone trial with bone endpoints has been undertaken, but there are two published trials of physiologic dose (not depot) cyclic medroxyprogesterone (MPA) of which we are aware, one a randomized controlled trial in healthy, normal weight, physically active women in their early 30 s [15] and one an open although apparently randomized trial in underweight teenagers with amenorrhea or oligomenorrhea [52]. The prospective trial of cyclic medroxyprogesterone acetate (MPA, 10 mg/day for 12 days a month) for underweight teenagers [52] is compared with the randomized, double-blind placebo-controlled two by two factorial design trial of cyclic MPA (10 mg/day for 10 days a month) and/or calcium supplementation (1000 mg/d) [15]. Women participating in this latter one-year trial differed from those in the teen study in being healthy, of normal weight, and physically active. In addition, they had a range of menstrual cycle and ovulatory disturbances including hypothalamic amenorrhea, oligomenorrhea, or ovulatory disturbances within regular cycles [15]. Bone change across one year was compared by randomization to cyclic MPA or to placebo [15]. 

Women with regular cycles were required to have two consecutive cycles with proof of ovulatory *disturbances* by QBT before enrolment. Participants were stratified by amenorrhea, oligomenorrhea, anovulation, and short luteal phase cycles into one of four groups—(1) cyclic MPA (10 mg for 10 days a month or cycle days 16–25) with active calcium (an additional 1000 mg/d); (2) cyclic MPA with placebo calcium; (3) placebo cyclic MPA with active calcium or (4) both MPA and calcium placebos [15]. The primary outcome, BMD of L1-4 in the spine, was measured at the beginning and the end of the year, as were body weight, height, and skin folds. Women also recorded 3-day diet diaries every three months and daily completed a Menstrual Cycle Diary [55] record daily, as well as recording their basal temperatures and exercise duration, type and mean exercise heart rates. 

Results in the 61 women completing this cyclic MPA trial showed that bone change over one year was positive in those assigned to cyclic MPA with or without calcium supplementation and averaged +1.7 ± 0.5 (SEM) percent (2 × 2 ANOVA *F* = 19.43, *P* = .0001). The effect of calcium supplementation was not quite significant (*F* = 3.34, *P* = .073); however it prevented some bone loss (mean change = − 0.7%, *P* = .28). Women assigned to both placebos lost bone at a significant rate (−2.0%, *P* = .005) despite being of normal weight, having regular exercise and adequate calcium intakes [15]. 

In a small open, apparently randomized (no clear RCT methodology provided) trial of underweight or anorexic teenagers with amenorrhea, who had inadequate calcium intakes (less than 1300 mg/d), the girls were assigned to cyclic MPA (10 mg for 12 days/month, *n* = 5), oral contraceptives (35 *μ*g ethinyl estradiol, *n* = 5), or placebo (*n* = 5) [52]. Young women in the same study who had oligomenorrhea were assigned to cyclic MPA (*n* = 5) or placebo (*n* = 4). Amenorrheic women on oral contraceptives appeared to gain spine BMD while those on cyclic MPA and placebo lost BMD [52]. However, these results are flawed by the differences of endogenous estradiol between oligomenorrhea and amenorrhoea as well as by the undernutrition of enrolled young women, and the few participants.

### 3.3. Progesterone and Bone in Perimenopause

#### 3.3.1. Bone Turnover Markers in Perimenopause

Although perimenopause is understood to be a time of dropping estrogen levels, its hormonal changes are much more complex than estrogen deficiency [10]. Hormonal perimenopausal changes involve altered control of gonadotrophins [56], disturbances of feedback of ovarian hormones at the pituitary and hypothalamic levels, at least partly through the inhibins [57], and erratic ovarian follicular growth despite decreasing numbers of follicles [58]. Ovulation disturbance is one of the consequences of these perimenopausal hormonal changes [59], which, in turn, may accentuate the changes. 

In the normal menstrual cycle, progesterone exerts feedback on the hypothalamic GnRH pulse generator and slows the frequency of GnRH pulses [60, 61]. However the amplitude of the pulses is higher during the luteal phase compared with the follicular phase. Towards the end of the luteal phase, the decreasing progesterone concentrations cause the GnRH-pulse generator to accelerate again. During these few days of acceleration, GnRH-receptors in FSH-producing cells are particularly sensitised, so that, for a few days before, during and after flow, FSH-levels rise [62]. This mechanism is pronounced during perimenopause, when increasing numbers of anovulatory cycles go hand in hand with rising early follicular phase FSH levels.

This next section will first review several studies of cross-sectional BMD values and bone resorption markers related to pre-, peri-, and postmenopausal status (Table 2). Results of prospective changes in bone turnover markers in women who differed in reproductive status but were all over age 40 and changes in spinal BMD by QCT in untreated pre-, peri-, and early postmenopausal women at baseline, two, and six years [63] will be studied. 

The majority of studies on perimenopausal bone change have been conducted using dual (energy) X-ray absorptiometry (DXA) of the hip and/or spine. DXA provides an areal, rather than true volumetric summary measurement of mineral content including both cortical and trabecular bone. Cancellous or trabecular bone, which is more responsive to hormonal changes, and measured volumetrically by QCT, provides a more sensitive assessment of change in BMD within the more metabolically active cancellous compartment and may result in earlier detection of bone loss. 

Since perimenopause is characterized by unpredictable and unstable endocrinological changes, systematic comparison and classification are difficult. Efforts to establish standards for scientific comparisons and for clinical use have led to five phases of reproductive transition, based on endocrinological and clinical criteria defined in international boards such as the WHO Scientific Group [71] and Workshops (e.g., Staging of Reproductive Aging Workshop (STRAW) [72]), with the aim of achieving comparability of scientific results on perimenopause. To date international standardisation has not been achieved, and the newly defined criteria are still not used consistently. Therefore publications reporting that they studied “perimenopausal women” may include them either with premenopausal or postmenopausal groups, as by Kushida et al. or Melton et al. in Table 2 [66, 68], or mix them with others to form a group of middle-aged women such as ages 40–59 years [65] without applying any distinct hormonal or menstrual cycle criteria. Another way of dealing with the problem of definition has been to take change from premenopause to postmenopause over a time course of many years, which is only possible in studies such as those published by Löfman or Ravn [67, 69] (5-year follow-up, see Table 2). These studies, however, carry the risk of not capturing perimenopause itself over such long intervals.

Amongst the published cross-sectional data, only Ebeling et al. [64] and Sowers et al. [70] studied truly perimenopausal groups of women. Ebeling found elevated bone resorption rates and declining bone density in 118 perimenopausal women. Sowers, in the baseline data of the multiethnic participants (*n* = 2336 women aged 42–52) from SWAN, the Study of Women Across the Nation, also showed that increased bone turnover begins years before menopause [70].

A meta-analysis of within-centre studies documenting both perimenopausal and postmenopausal rates of spinal bone loss earlier showed significantly greater rates of loss in perimenopause (−1.8 versus −1.2%/year) [10]. That analysis reported preliminary Melbourne Midlife Women's Health study results prior to their publication [73]. Using DXA, this study showed that spine bone loss was increased during perimenopause. However, in 224 untreated pre-, peri, and postmenopausal participants (*n* = 78 early perimenopausal, *n* = 12 late perimenopausal), the greatest amount of loss occurred in the first three years following the final menstrual flow [73]. This may have been because of inclusion of the first year after the final menstrual flow in “postmenopause” rather than perimenopause [59], or because of the relative time lag of DXA for bone changes affecting mainly or exclusively the trabecular compartment. Accordingly, early cycle elevated FSH and low estradiol values, as are commonly found in postmenopause, correlated with this increased loss [73].

 Another study by Slemenda et al. showed increased bone loss in 62 perimenopausal women from a total of 231 untreated women. Apart from low estrogen, bone loss in this study was also associated with lowered serum androgen levels [74]. The Michigan Bone Health Study cohort (513 women, aged 25–45 randomly sampled from the population) documented that DXA of the spine was three percent lower in perimenopausal than in premenopausal women and that annual bone loss was significantly elevated in perimenopausal women, when compared with premenopausal participants [75]. These observations were confirmed by the even larger SWAN study (*n* = 2311) [76]. In the first 4 years of this study with annual bone density measurements and early follicular phase serum hormone values, this study showed that in both baseline and follow-up, elevated FSH levels were associated with decreased bone density, while estradiol was not [76]. In both the Australian and USA large studies, however, hormone values were only taken during the early follicular phase, a time when FSH is often elevated in perimenopausal women, estrogen is normally low, and progesterone cannot be evaluated. So, despite the large size and the power of these studies, they could only assess hormonal effects of the first week of women's menstrual cycles, excluding the remaining 75% of potentially available information [70]. 

Two prospective observational German studies attempted to systematically characterize the changes in bone metabolism associated with perimenopause. In the first study, serial bone turnover marker measurements were made on 64 healthy women over age 40, who had taken no exogenous hormones and were within three reproductive life phases: premenopause (mean age 43.7 years, *n* = 20), perimenopause (mean age 50.3 years, *n* = 24), and postmenopause (mean age 52.2 years, *n* = 20) [78]. These prospective, serial bone marker measurements were first made on four visits across one year (0, 3, 6, and 12 months). Parameters relating to bone resorption were the urinary excretion of pyridinoline (PYD), deoxypyridinoline (DPD), and N-terminal telopeptide (NTX), all corrected for creatinine. As well, serum bone formation markers were measured including osteocalcin (OC) and bone-specific alkaline phosphatase (BAP) [78]. In these midlife women with regular cycles, this study found a significant decrease over time for the bone formation marker BAP, leading to the conclusion that the metabolic changes in bone remodeling commonly associated with perimenopause (like the higher estradiol levels and disturbed ovulation [10]) had already begun in the late reproductive phase [30]. These participants were followed for a second year [79], as well as eventually over six [63] and nine years [80]. 

The six-year follow-up allowed for a longitudinal comparison of bone changes in pre- as opposed to perimenopausal and early postmenopausal women. Analysis of QCT changes over time required classification of women's reproductive status and its changes over time. Invariably for all analyses, the perimenopausal period—during which estrogen levels were still adequate—was associated with the greatest reduction of QCT, with loss rates reaching 6.3%/year. Total bone loss differed by pattern of individual women's experiences of the transition from pre- to postmenopause (one-way ANOVA *P* < .05); the average rate of loss was slower in the early postmenopausal years [63].

**Figure 4:**
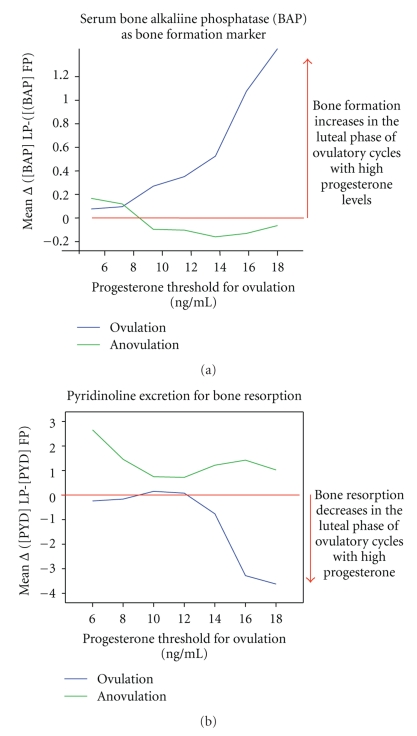
Intra-cycle follicular-luteal phase change in two different bone turnover markers by the serum progesterone level used as a threshold for ovulation. (a) shows the bone formation marker bone-specific alkaline phosphatase (BAP) in serum, (b) depicts changes in the bone resorption marker Pyridinoline (PYD) extracted with HPLC from urine and normalized to creatinine reprinted from [77]. Permissions provided.

The second prospective observational study was initiated to further explore the drop in BAP observed in the first study and to systematically monitor bone turnover markers in the follicular and luteal phases of serial cycles in 8 women whose averaged age was 46 years. From a total of 170 cycles, 84 cycles with luteal phase serum sampling were analysed. Categorical differences were calculated to detect individual intracycle changes in bone metabolism. Figures 4(a) and 4(b) show the within-cycle change (luteal minus follicular phase levels) of serum BAP (Figure 4(a)) or HPLC-extracted urinary pyridinoline (PYD; Figure 4(b)) by the threshold progesterone level used to document ovulation [81]. These two figures show that both PYD and BAP patterns differ in ovulatory and anovulatory cycles. Further, given that positive values mean increases in the luteal phase and negative values mean luteal phase decreases, these data suggest that the higher the progesterone level the more bone formation. As mentioned earlier, decreased bone resorption markers in the luteal phase occur because resorption continues to be controlled by moderate luteal phase estradiol levels.

Seifert-Klauss and colleagues are currently conducting the ongoing “PENO-Study” (Perimenopausale Knochendichte [bone density] und Ovulation), which is a prospective observational study over the course of two years. Its purpose is to investigate menstrual cycles, hormonal values, bone turnover markers, and changes in bone mineral density (BMD) during the perimenopause to answer the following question: do perimenopausal women with a higher rate of anovulatory cycles have increased loss of bone density?

Inclusion criteria for this study are age >45 years, cycle lengths no greater than 42 days, no use of exogenous hormones during the 6 months prior to study onset, and no medical reasons for low bone mass. Lumbar spine trabecular BMD measurements are performed using QCT at baseline and after two years. Participants note the beginning and end of each cycle and used a cycle monitor to detect the day on which there was a high probability of ovulation. 

**Figure 5:**
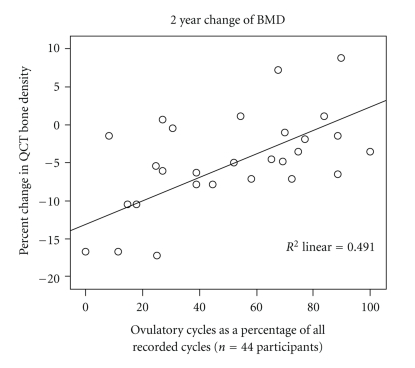
The 2-year-change of trabecular lumbar spine bone mineral density documented by Quantitative Computed Tomography (QCT) is shown by rate of ovulatory cycles in 28 women with complete ovulation data out of the 44 women studied prospectively in the ongoing PEKNO-Trial. Assessed by a commercially available ovulation monitor device, ovulation-likelihood was verified by luteal phase serum sampling. The graph illustrates the significant linear relationship (*r* = 0.7; *P* < .05) observed between the percentage of ovulatory cycles and BMD loss in pre- and perimenopausal women. This figure is from a presentation on the interim analysis by T. Wimmer and V. Seifert-Klauss to the Congress of the German Menopause Society (Deutsche Menopausen Gesellschaft) in Hamburg, November 6th 2009 (unpublished). The authors provide permission.

Results are available so far for 54 women (mean age 48.3 ± 2.3 SD years) who have recorded 673 evaluable cycles and had 132 luteal phase blood tests suitable for analysis. QCT measurements at baseline show that 45 women had normal bone density (mean 148.2 ± 19.3 mg Calcium-Hydroxyapatite (Ca-HA)/mL^3^), while nine had osteopenia (mean 103.7 ± 7.3 mg Ca-HA)/mL^3^. Women with normal BMD at the beginning of the study, and those who maintained BMD levels within the normal range over two years, were more likely to experience normal ovulatory cycles with fewer ovulatory disturbances than those women whose initial or two-year QCT values showed osteopenia. During the course of this two-year study, the proportion of ovulatory cycles related to QCT bone change (*r* = −0.7, *P* < .05) (Figure 5). Also, as has previously been shown [59, 82], progesterone levels decreased before cycles without ovulation became common [77]. 

Although women are known to lose bone rapidly *before* as well as after they become postmenopausal [10, 83], this study adds to existing data by showing that this bone loss is not simply due to the increased bone resorption caused by the perimenopausal swings in estrogen levels but is also related to progesterone levels and ovulatory characteristics.

### 3.4. Progesterone and Bone in Postmenopausal Women

Postmenopausal women are more likely to experience fragility fractures than are pre- or perimenopausal women. This increased fracture risk is usually ascribed to estrogen deficiency—but this state also includes progesterone “deficiency.” A number of published investigations have asked two questions about the relationship of progesterone to bone change in postmenopausal women. These research questions are as follows. (1)* Does progesterone therapy prevent or treat osteoporosis in postmenopausal women?* And, (2)* Does progesterone therapy add to the bone-positive effects of anti-resorptive therapies (such as estrogens, calcitonin, or bisphosphonates)?* This section and the next will review the available human data to answer these two questions.

#### 3.4.1. Progesterone Therapy for Postmenopausal Osteoporosis

To determine whether progesterone is effective treatment for osteoporosis in postmenopausal women, changes in BMD by DXA and/or QCT in four RCTs compared treatment with progesterone or MPA with placebo. Gallagher, in the earliest study, asked whether a high dose of the non-androgenic progestin, medroxyprogesterone [MPA] effectively treated postmenopausal osteoporosis. The 20 mg dose of MPA for 23 of 28 days did not prevent bone loss by spine dual photon absorptiometry (DPA) [84]. Likewise, Table 3 shows bone change in two further RCTs of more standard MPA doses of 10 mg/d, 300 mg/d of oral micronized progesterone (OMP), or 20 mg/d of progesterone cream. The net result of the placebo-controlled trials with MPA was bone loss (−2.2% per year) despite these MPA or progesterone therapies and with no apparent difference from the bone change on placebo (−2.4%/year). 

One of the RCTs of MPA alone and bone change was a unique one-year randomized blinded comparative study versus conjugated equine estrogen (CEE) in 41 premenopausal women who had just undergone premenopausal abdo-minal hysterectomy with bilateral ovariectomy for benign problems [85]. CEE (0.6 mg/day) was compared with MPA (10 mg/day) over one year [85]. All 41 women began study participation after fasting blood and urine samples were obtained on the morning they were discharged following their surgery [85]. Results showed highly significant rates of spinal cancellous QCT bone loss in women on both therapies (−15% MPA, −8.3% CEE, *P* = .04). MPA also did not prevent significant bone loss in the whole body (−2.8%) and femoral neck (−5.2%). 

The results of this MPA versus CEE randomized comparative trial may provide clues to causes for major bone loss despite progesterone/MPA therapy with their osteoblast differentiating and bone formation effects. On average at seven days following surgery, bone resorption markers were three to five *standard deviations* higher than premenopausal levels. These bone resorption markers did not decrease across a year of MPA therapy despite the fact that all women were supplemented with 600 mg of additional calcium/day and gained approximately 2.5 kg in weight [85]. These data suggest that MPA does not decrease bone resorption. One further randomized 2-year placebo-controlled trial of a progestin and bone change is available. This study treated early postmenopausal women with promegestone, a 19 nor-progestin, or placebo and showed some prevention of bone loss (−1.3% versus −4.5% on placebo, *P* = .05) [88]. Urinary calcium excretion was significantly decreased in the promegestone group, however, suggesting it may decrease resorption rather than acting through the progesterone receptor. 

Taken together from this meta-analysis, the answer to the first question is as follows *MPA/Progesterone alone is not effective therapy for postmenopausal osteoporosis* because it has no or little effect to control bone resorption, the driving force in human bone loss and osteoporosis.

#### 3.4.2. Progesterone as Co-Therapy with Antiresorptives for Postmenopausal Osteoporosis

Yearly percent bone change on co-therapy with an antiresorptive and progesterone or MPA compared with the antiresorptive agent alone has been studied in five randomized, double, blind controlled trials to answer the second research question named above (Table 4). Two of these were major studies in the bone field (Postmenopausal Estrogen Progestin Investigation [PEPI] [89] and the Women's HOPE trial) [90]. A more recent study combined MPA 10 mg/d with oral micronized estradiol in a dose of 1 mg/d, versus the estradiol alone [87]. The results of these RCTs show the mean change in spinal BMD on co-therapy with daily low-dose MPA, and an antiresorptive was slightly more positive (+1.7%/year) than with the antiresorptive alone (+1.3%/year), a difference of about 24%. In both of the largest studies [89, 90], estrogen-progestin spine results were noted to be significantly more positive than those related to the antiresorptive alone [89, 90]. This, however, did not appear to occur when the progestin was given cyclically [91], suggesting that daily progesterone is needed to increase BMD in menopausal women. Therefore the answer to the second question is as follows. *Progesterone daily co-therapy with estrogen is more effective than estrogen alone for postmenopausal osteoporosis.*

The non-randomized clinical studies combining MPA with an antiresorptive therapy show similar but even greater increases in BMD on MPA and antiresorptive co-therapy compared with the antiresorptive alone [92–94]. It is very difficult to compare studies in women who have had hysterectomy with/without ovariectomy because women with hysterectomy, and certainly following ovariectomy, are much more likely to be treated with estrogen than are women with natural menopause; this represents confounding by indication. The New Zealand studies examined older postmenopausal women with high rates of bone loss who were treated with CEE plus 5 mg/d of MPA or with CEE alone—this one-year study saw a 65% greater spine BMD increase on combined therapy (6.6%) compared with CEE alone (4.0%) [93]. A prospective study also from New Zealand and in a similar population looked at the rates of bone change in the hip and spine when either CEE or transdermal estradiol was paired with MPA [92] and compared with placebo. One-year results were quite positive in the spine (+7.1%) and in the femoral neck (2.9%) but there was no anti-resorptive alone comparison in this descriptive study. Finally, a small pilot study on a random sample of (*n* = 20) clinical patients treated with MPA combined with an early bisphosphonate (intermittent cyclic etidronate, Didrocal) compared their bone change data with that from a meta-analysis of RCTs of etidronate alone [95]. In this comparison, spine increases were greater on MPA-antiresorptive co-therapy (+2.6%) than on antiresorptive therapy alone (+1.8%); femoral neck BMD increases on MPA co-therapy were also more positive (+1.5% versus +0.5%) [94]. 

So far in this paper, the fracture prevention shown in the CEE-MPA and the CEE only arms of the Women's Health Initiative (WHI) trials has not been discussed [5, 96]. These are not only the largest studies of bone in women randomized to postmenopausal hormone therapy or placebo (*n* = 27,000 in both studies together), they are also the only randomized, placebo-controlled studies to show fracture prevention with ovarian hormone therapy. These data are especially important since the study population was not selected for low bone mass or osteoporosis risk factors. Ideally we could compare the rates of fracture in the co-therapy with the CEE-only arms but this is not possible because each was in a different randomization scheme and had its own placebo group. We have requested collaboration with WHI investigators in such an adjusted analysis.

#### 3.4.3. Breast Cancer and Other Issues Relating to Progesterone/MPA Therapy in Postmenopausal Women

Data so far suggest that progesterone or MPA co-therapy with estrogen is more effective for osteoporosis; however breast cancer is an important clinical concern [96]. Combined hormone therapy containing MPA has been repeatedly associated with increased breast cancer risk [97–99]. The prospective E3N observational study, following 80,000 French women over eight years however, saw an increase in breast cancer risk only with estrogen alone or with synthetic progestins, but not with oral micronized progesterone combined with estrogen [99, 100]. Randomized controlled research on the breast cancer risks with estradiol plus progesterone is needed. Likewise, effects of progesterone and various progestins on intramammary estradiol metabolism are needed before combined estradiol and progestin would be considered safe or acceptable. 

Other important non-bone issues in women with postmenopausal osteoporosis are sleep disturbances and hot flushes and night sweats (vasomotor symptoms, VMS) that have been linked to increased bone loss [101–103]. Oral micronized progesterone is clinically useful for insomnia and in a dose of 300 mg at bedtime significantly increased total sleep time, decreased time required to fall asleep, and increased early night REM sleep in a cross-over RCT [104]. That trial also showed that, after 21 days of treatment, progesterone caused no lack of alertness or any cognitive impairment in the morning [104]. Vasomotor symptoms, that occur in about 70% of postmenopausal women and are severe in almost 10 percent, are effectively treated by MPA [105, 106] and also by oral micronized progesterone treatment [100].

### 3.5. Conclusions

Although the dominant osteoporosis paradigm for women is, and should remain, centred on estrogen, progesterone is emerging as an important partner hormone that collaborates with estrogen. *In vitro* studies of human osteoblasts in culture, prospective studies in adolescent, premenopausal, perimenopausal, and postmenopausal women all indicate that progesterone—likely working through bone formation pathways—plays an active role in maintaining women's bone and in osteoporosis prevention. 

Finally, although progesterone or MPA therapy does not prevent bone loss when bone turnover is high, evidence from a number of randomized controlled trials suggests that progesterone as co-therapy with an antiresorptive agent may have promise. Data on progesterone co-treatment and fracture prevention are urgently needed, as is more information about the microarchitectural and histomorphometric changes during progesterone therapy. 

Progesterone, a physiological ovarian steroid that is normally secreted in high levels for two weeks per menstrual cycle in ovulatory menstruating women, appears to have complementary bone actions with estrogen and antiresorptive therapies. Progesterone deserves to be studied more as a new and emerging agent for achieving and preserving peak bone mass, for prevention of pre- and perimenopausal bone loss, and, with an antiresorptive therapy, in increasing BMD and potentially decreasing fractures in postmenopausal women. 

## Figures and Tables

**Table 1:** Prospective studies of spinal Bone Mineral Density (BMD) change by ovulatory menstrual cycles compared with ovulatory disturbances (anovulation and short luteal phases within normal length cycles). BMD is by Quantitative Computed Tomography (*) or Dual Energy X-ray Absorptiometry (^+^). All data are shown as mean ± SD.

Manuscript	Number women	Duration (years)	Age ± SD (range)	Body Mass Index	# Cycles/year	Cycle length (days)	% Bone change/year-spine	
							Normal^∙^	Ovulatory disturbances
Prior 1990 [15]	66	1	33.7 ± 7.1 (20–42)	22.0 (18–24.9)	10 (6 to 13 cycles)	28.2 ± 2.6	(*) +0.2	(*) –3.3
Prior 1996 [51]	27	4	35.9 ± 4.9	21.7 (18–24.9)	1.5 (3–46 cycles)	27.8 ± 2.4	(*) *n* = 14 −0.98°	(*) *n* = 13 − 0.94°
Waller 1996 [52]	53	1.5	33.4 ± 4.3	$\text{NR}\hat{\;}\;$	2.7	$\text{NR}\hat{\;}\;$	(^+^) −0.05	(^+^) +0.55
Waugh 2007 [53]	189	2	32.4 ± 4.6 (21–40)	24.3 (range not given)	5	28.9 ± 3.9	(^+^) +1.6	(^+^) −0.4
Bedford 2010 [48]	123	2	22.1 ± 3.3 (19–35)	21.8 ± 2.5	6.8 ± 7.0	30.8 ± 4.1	(^+^) +1.9	(^+^) +0.7

Totals (Mean)	458	2.1	31.4	22.5	6.6	28.9	+0.53	−0.68

°Based on a median split of % all cycles with ovulatory disturbances. “Normal” = 0%–33% of all cycles with ovulatory disturbances and “Ovulatory Disturbances” = 34%–100% of cycles with ovulatory disturbances.

^∙^“Normal" means normal menstrual cycle length with ovulation and a normal luteal phase length

# Numbers of cycles/year in which ovulation and ovulatory disturbances as well as cycle length were documented.

$\hat{\;\;}\text{NR}$ means not recorded.

**Table 2:** Cross-sectional studies on perimenopausal bone metabolism and bone mineral density.

Author	Title	Design	Methods	Relevant findings	Conclusion
Ebeling et al. 1996 [64]	*Bone turnover markers and bone density across the menopausal transition*	281 women, 45–57 years. 3 groups: 60 premenopausal 118 perimenopausal 103 postmenopausal (of these, 36 with HRT)	DXA E2, FSH, LH, inhibin on day 4–8 of menstrual cycle if applicable Bone formation: OC, BAP, PICP Bone resorption: PYD, DPD, NTX	Postmenopausal group: BMD ↓ ↓. Loss in BMD correlated with age in perimenopausal group. Perimenopausal group: LH, FSH doubled versus premenopause; E2, BAP did not differ between premenopausal and perimenopausal group. ↑ PYD, DPD, NTX, BAP, and OC in postmenopausal versus premenopausal group. perimenopausal group: Positive correlation of BMD with DPD. All women: correlation of BMD with NTX, BAP, OC, FSH	Perimenopause: increased bone resorption rate and decreased bone density. Other factors apart from E2 are involved in the development of postmenopausal osteoporosis

Khosla et al. 2005 [65]	*Relationship of volumetric bone density and structural parameters at different skeletal sites to sex steroid levels in women*	235 untreated women 3 groups: (i) premenopausal (20–39 years.) (ii) mixed (40–59 years.) (iii) postmenopausal (>60 years.)	QCT E2, Testosterone	Postmenopausal group: significant correlation of low bioavailable E2 and BMD (trabecular and cortical). 40–59 years: significant correlation between average rise in bioavailable E2 and loss in trabecular BMD.	Trabecular bone reacts faster to lowering E2. The threshold for estrogen deficiency in cortical bone in women appears to be lower than that in trabecular bone.

Kushida et al. 1995 [66]	*Comparison of markers for bone formation and resorption in premenopausal and postmenopausal subjects, and osteoporosis patients*	95 premenopausal women, 30–53 years. 66 postmenopausal women, 50–69 years 29 untreated women with osteoporosis, 55–91 years. No distinct perimenopausal group, but included in pre- and postmenopausal	No BMD measurement Bone formation: AP, OC, PICP, Bone resorption: PYD, DPD	Postmenopausal group: AP, OC, PICP, PYD, DPD significantly higher than in premenopausal group. In osteoporosis: PICP, PYD, DPD significantly higher than in postmenopausal group. Women ≥ 50 years: PYD, DPD higher than in women 30–49 years.	Markers in postmenopause higher than in premenopause. In women with osteoporosis resorption markers are higher than formation markers.

Löfman et al. 2005 [67]	*Common biochemical markers of bone turnover predict future bone loss: a 5-year follow-up study *	Cross sectional study (+ longitudinal) 192 women, 21–79 years. 3 groups (i) premenopausal (ii) perimenopausal (i.e., premenopausal at baseline and postmenopausal after 5 years). (iii) postmenopausal	2x DXA Bone formation: BAP, OC, AP Bone remodelling: Hpr Ca	Baseline values of markers correlated negatively with baseline BMD. AP, OC, Hpr, Ca rise at the “beginning of menopause” 15 years after menopause: OC and Hpr are still elevated.	Bone markers and current BMD could give information about coming loss of BMD.

Melton et al. 1997 [68]	*Relationship of bone turnover to bone density and fractures*	351 women, 20–80 years. 2 groups: 138 premenopausal 213 postmenopausal (i) 47 with HRT (ii) 166 without HRT of these, 89 cases of osteoporosis No distinct perimenopausal group	DXA Bone formation: OC, BAP, PICP Bone resorption: PYD, DPD, NTX	Premenopausal group: OC, NTX negatively correlated with BMD. Postmenopausal group: increase of markers with age. Postmenopausal group: OC, BAP, NTX, PICP negatively correlated with BMD. Osteoporosis: markers↑, BMD↓.	Combination of markers with BMD measurement is sensible for prediction of individual fracture risk. NTX is best predictor of loss in BMD.

Ravn et al. 1996 [69]	*High bone turnover is associated with low bone mass in both pre- and postmenopausal women*	979 women, 30–75 years. 2 groups: 334 premenopausal 645 postmenopausal 5 year-longitudinal analysis. No distinct perimenopausal group, but included in pre- and postmenopausal	DXA Bone formation: OC, AP Bone remodelling: HydroxyProline Bone resorption: CTX	Premenopausal <50 years: markers stable. Women with highest markers had significantly lower BMD. OC and CTX correlated with BMD. Postmenopausal group: CTX, OC sign. higher than in premenopausal group. 5 years. after menopause: CTX, OC stable again.	Bone metabolism is accelerated in perimenopause and early postmenopause.

Sowers et al. 2003 [70]	*The association of endogenous hormone concentrations and bone mineral density measures in pre- and perimenopausal women of four ethnic groups: SWAN*	2336 women, multiethnic, 42–52 years. 2 groups: (i) premenopausal (ii) perimenopausal	DXA E2, FSH, T, DHEAS, SHBG (day 2-7 of menstrual cycle if applicable)	Perimenopausal group: FSH higher and BMD lower than premenopausal group. All women: negative correlation of FSH with BMD. No correlation of E2 and BMD.	Loss of BMD starts before menopause.

**Table 3:** Double-blind randomized controlled trials of percentage spine Bone Mineral Density (BMD) change per year in postmenopausal women treated with Progesterone/Medroxyprogesterone (MPA) compared with placebo.

Author/year and reference	Total number	Age	Bone site	Years	Drug and dose	Schedule	Number	% BMD Change (Active)	Number	% BMD Change (Placebo)
Gallagher 1991 [84]	81	51.7 ± 4.4	DPA* L2−4	2	MPA 20 mg	23/28 days	20	Spine −2.5	20	Spine −3.8
			Radius				20	Radius 0.0	18	Radius −2.4
										
Prior 1997 [85]	33	45 ± 5	QCT T12−L3 DXA	1	MPA 10 mg	Daily	18	QCT −15	NA^∙^	NA
			WB+					WB −2.8	NA	NA
			FN++					FN −5.2	NA	NA
										
Leonetti 1999 [86]	102	52.5	DXA L2−4	1	*P_4_ Cream 20 mg	Daily	43	Spine −1.4	47	Spine −1.0
			T Hip				43	T Hip −2.5	47	T Hip −1.0
										
Liu 2005 [87]	132	52.5	DXA L2−4	2	^+^OMP	300 mg Daily	15	Spine −1.0	23	Spine −1.0
			FN				15	FN −0.5	23	FN −0.0
					MPA	10 mg Daily	16	Spine −1.9	23	Spine −1.0
							16	FN −1.1	23	FN −0.0

Mean % Change					OMP & P_4_ Cream		58	Spine −1.2	70	Spine −1.0
					MPA		36	Spine −2.2	43	Spine −2.4

*P_4_: progesterone

^+^OMP: oral micronized progesterone

^∙^NA: not available—this trial was controlled by conjugated equine estrogen and without a placebo.

**Table 4:** Comparison of randomized double-blind controlled trials of bone change in postmenopausal women with osteoporosis comparing combined estrogen [Conjugated Equine Estrogen (CEE) or Estradiol (E_2_)] plus progesterone or medroxyprogesterone (MPA) and documenting percentage (%) change per year in Bone Mineral Density.

Author	Type	Number	Age ± SD	Bone sites	Years	Anti-resorptive mg/d	Progesterone/ MPA mg/d	Number	Combined % bone change	Number	Anti-R* % bone change
Gallagher 1991 [84]	RCT not blinded	81	52 ± 4	DPA L2−4 SPA	2	0.3 CEE 23/28 days	MPA 10 mg 23/28 days	16	Spine +0.25	18	Spine +1.0
				Radius					Radius +0.0		Radius −0.05
											
PEPI 1996 [89]	DB-RCT	875	56	DXA L2−4	3	CEE 0.625	MPA 2.5 mg/d	174	Spine +1.6	175	Spine +1.4
				Total Hip (TH)				174	TH +0.5	175	TH +0.5
											
Adachi 1997 [91]	DB-RCT	98	54	DPA	1	CEE 0.625	MPA 10 mg for 15 d/mo.	33	Spine +2.7	34	Spine +1.9
											
Lindsay 2002 [90]	DB-RCT	695	58	L2−4 DXA	2	CEE 0.625	MPA 2.5 mg/d	81	Spine +1.7	84	Spine +1.2
				Total Hip (TH)					TH +1.3		TH +1.4
						CEE 0.45	MPA 2.5 mg/d	87	Spine +1.5	91	Spine +1.1
									TH +1.1		TH +1.0
											
Liu 2005 [87]	DB-RCT	132	53	DXA L2−4	2	E_2_ 1 mg/d	MPA 10 mg/d	20	Spine +2.3	23	Spine +1.3
				FN		OME^#^			FN +0.9		FN +1.0

$\text{Totals}\hat{\;\;}$								330	Spine +1.7	425	Spine +1.3

^∙^DPA: dual photon absorptiometry, SPA: single photon absorptiometry, DXA: dual energy X-ray absorptiometry.

*Anti-R: antiresorptive therapy, ^#^OME: oral micronized estradiol.

$\hat{\;\;}$Note that the two estrogen-dose arms of the Lindsay study were considered as two different studies in the mean spine bone change.
